# Novel Aortic Root Measurement Technique Using the Laplace Diameter for Identifying Patients at Risk for Type A Dissection

**DOI:** 10.1016/j.atssr.2025.06.032

**Published:** 2025-08-15

**Authors:** Asanish Kalyanasundaram, Lisa C. Harling, Mohammad A. Zafar, Hesham Ellauzi, Bulat A. Ziganshin, John A. Elefteriades

**Affiliations:** 1 Aortic Institute, Yale University, New Haven, Connecticut; 2 Department of General, Visceral, Vascular and Transplant Surgery, Otto von Guericke University Medical School, Magdeburg, Germany

## Abstract

**Background:**

The Laplace law is commonly applied to calculate aortic wall stress by using the luminal pressure and the aortic diameter. Wall stress bears on the likelihood of aortic dissection in dilated aortas. However, the Laplace law applies only to circles and cylinders. It is not applicable for the aortic root, which can be more closely described as a cloverleaf shape, rather than a circle. We have recently developed a mathematically based measuring technique specifically for the aortic root. This *Laplacediameter* provides an appropriate means to measure a “diameter” for the cloverleaf shape of the aortic root.

**Methods:**

In this study, we assessed the predictive ability of the Laplace diameter vs the standard sinus-to-commissure measurement in 33 patients who underwent predissection computed tomographic scans for unrelated reasons in close temporal proximity to their acute aortic event. We analyzed 14 chest computed tomographic scans of 33 patients who received predissection scans for unrelated reasons.

**Results:**

We observed a 16.1% increase in the mean root diameter using the Laplace diameter. We found that 21.4% of the analyzed predissection scans could have resulted in detection and prevention of the aortic dissection through surgery if the Laplace diameter had been applied.

**Conclusions:**

We validated the novel method of the Laplace diameter clinically in determining the aortic root diameter and detecting the risk of aortic dissection.

**In Short**

▪ Using the Laplace measurement for the aortic root may increase detection of patients at risk of aortic type A dissection.
▪ Decreasing the surgical threshold from 55 mm to 50 mm increases the detection rate of patients at risk of aortic type A dissection.
▪ This study retrospectively validated the Laplace diameter using predissection computed tomographic scans and demonstrated its effectiveness in identifying high-risk patients.

The Laplace law describes the tension in vessel walls as influenced by the luminal pressure and the diameter (wall tension [T] = blood pressure [BP] × circumscribing circle diameter [d]/[2 × aortic wall thickness]).^1^ This leads to larger vessels experiencing greater wall stress than their smaller counterparts. This classical law, however, is only applicable in ideal cylinders (with circular cross-sections). The aortic root is not cylindrical, bearing the well-known 3 aortic sinuses, which deviate markedly from a circular cross-section.

Aortic dissection is a lethal disease, with aortic dilation as its predecessor, but commonly a lack of other preliminary symptoms. Recently, root dilatation has been shown to be even more dangerous than a corresponding level of dilatation in the ascending aorta.^2^ Radiographic monitoring is therefore a key element in the prevention of aortic dissection. The 2022 American guidelines and the 2024 European guidelines recommend surgery in good-risk patients with diameters of ≥50.0 mm.^2^, ^3^, ^4^

However, determining the “diameter” of the aortic root is challenging because, unlike other segments of the aorta (ascending, arch, descending, abdominal), the aortic root resembles a cloverleaf shape rather than a cylinder (Figure 1).^1^ The measurement most commonly used, sinus-to-commissure, can lead to underestimation of the aortic wall tension and therefore the risk of aortic dissection. The alternate, commonly used sinus-to-sinus technique is similarly unsuitable.

**Figure 1:**
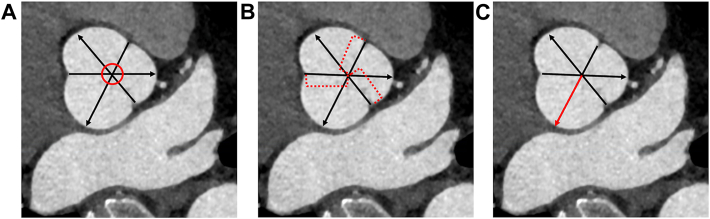
Visualization of the measuring technique used to obtain the Laplace diameter. (A) Placement of the 3 lines connecting commissures to the opposing sinus, intersecting in 1 point (red circle). (B) The distance from the intersection to each sinus is measured (dotted lines). (C) The longest distance becomes the Laplace radius, which is doubled to receive the Laplace diameter (red and black arrow).

Our new, biomechanically optimized measuring technique, referred to as the *Laplace diameter* of the aortic root represents the product of a mathematically derived extended law of Laplace, appropriate for clinical evaluation of the aortic root.^1^

Abundant evidence from our group and from the University of Pennsylvania group has shown that the thoracic aorta grows abruptly at the moment of aortic dissection, by ∼8 mm.^5^, ^6^, ^7^, ^8^ This has been a major factor motivating the “left shift” in guidelines for surgical intervention.^7^^,^^8^

## Material and Methods

This retrospective study was approved by the Yale University Human Investigations Committee. In this study, the Laplace diameter method was retrospectively applied to measure the aortic root on predissection chest computed tomographic (CT) scans of patients who sustained a type A dissection promptly subsequent to the incidentally performed CT scan. Of 212 acute ascending aortic dissection patients in our database, 33 had, by chance, undergone a predissection CT scan for unrelated clinical reasons, 14 of whom had scans with sufficient technical quality for precise measurements to be made.

The Laplace diameter of the aortic root was measured by using our newly published technique (Figure 1, Supplemental Video); specifically:

1. The viewing plane is rotated in the axial, sagittal, and coronal axes to visualize the aorta exactly perpendicular to its long axis at the level of the aortic root.
2. Lines are drawn connecting the midpoint of each sinus to the commissure directly opposite, always intersecting in 1 point (Figure 1A).
3. A measurement is taken from the intersection point of these 3 lines (“center”) to the midpoint of the corresponding sinus (Figure 1B).
4. The furthest distance from the center to a sinus is established (“Laplace radius”) (Figure 1C).
5. This is doubled to get the Laplace diameter.

The application of the new Laplace diameter measurement technique to patient CT scans is demonstrated in the Supplemental Video, which is reproduced with permission from Ban and colleagues.^1^

The standard sinus-to-commissure measurement and the Laplace diameter were both measured using the selected CT scans, and the resulting values were compared. We also calculated the less commonly used sinus-to-sinus measurements.

## Results

**Figure 2:**
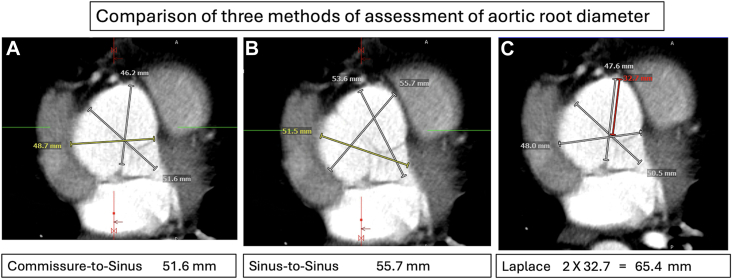
Comparison of aortic root measurements (gray lines) between the 3 techniques. (A) Commissure-to-sinus measurement. (B) Sinus-to-sinus measurement. (C) Laplace measurement. For the Laplace measurement, we take the largest radius (to the deepest sinus from the “center” intersection of lines) and multiply by 2 to convert to a Laplace diameter equivalent. Note the very large size of the right sinus, which is reflected in the Laplace measurement. The red line in the Laplace figure measures the largest radius, which is then doubled to calculate the Laplace diameter.

Predissection CT scans for 14 patients were of good enough quality to be evaluated. For this retrospective study, we measured the aortic root by the standard sinus-to-commissure technique, by the sinus-to-sinus technique, and by our Laplace diameter method. Figure 2 illustrates the 3 techniques applied to 1 patient. The mean root diameter using standard techniques was 42.8 mm (sinus-to-commissure) and 45.2 mm (sinus-to-sinus), whereas the mean Laplace diameter was 49.7 mm (16.1% increase for sinus-to-commissure and 9.96% increase for sinus-to-sinus).

If we consider the criterion for preventative predissection surgery of 55 mm, which is the commonly used cutoff point for patients not undergoing concomitant cardiac surgery or presenting with preexisting conditions affecting the aorta at the time of the patients’ predissection scans, none of the patients would have qualified for surgery if the sinus-to-commissure measurement were used. This corresponds to the clinical course of the patients because none of them received surgery to correct the dilation of the aorta.

**Figure 3:**
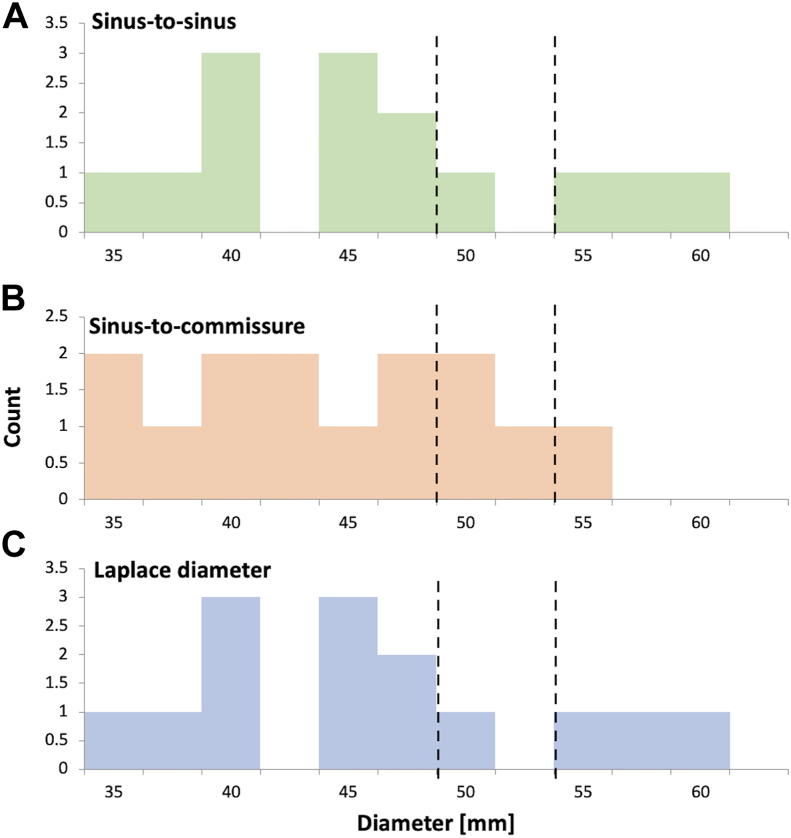
Histogram displaying the distribution of (C) Laplace diameter measurements compared with the distribution of other commonly used techniques ((B) sinus-to-commissure and (A) sinus-to-sinus). Surgical thresholds of 55 mm and 50 mm are marked.

Using the Laplace diameter to measure the aortic root, however, would have resulted in 3 patients qualifying for preventative surgery for aortic dilation greater the traditional 55 mm cutoff (at Laplace diameters of 58.4, 61.0, and 62.0 mm). Accordingly, 3 of 14 dissections (21.4%) would have been prevented by surgery and the patients potentially saved from this catastrophic complication (Figure 3).

If we consider the new, societal lower cutoff of 50.0 mm, 2 patients would have qualified for surgery with the standard sinus-to-commissure measurement (at 50.2 and 52.9 mm). Comparing the Laplace diameter with this, 9 patients would have been eligible to receive surgery (350% increase) at 50.0, 50.0, 50.2, 50.0, 50.4, 51.6, 58.4, 61.0, and 62.0 mm, preventing 7 of 14 dissections (50%) from occurring (Figure 2).

In the Table, we present the dimensions of the 14 patients, measured by sinus-to-commissure, by sinus-to-sinus, and by the Laplace technique. The average diameters are smallest by sinus-to-commissure (42.8 mm), intermediate by sinus-to-sinus (45.2 mm), and largest by the Laplace technique (49.7 mm). As seen in the Table, the average increase in diameter by applying the Laplace technique compared with the sinus-to-sinus was 4.5 mm; the increase compared with the sinus-to-commissure measurement was 6.9 mm.

**Table:** Comparison of Measurements on the Same Computed Tomographic Scans, Done by the 3 Techniques^a^

Patient	Sinus-to-Commissure (mm)	Sinus-to-Sinus (mm)	Laplace Diameter (mm)	Difference (Laplace—Sinus-to-Commissure) (mm)	Difference (Laplace—Sinus-to-Sinus) (mm)
1	34.0	30.6	36.4	+2.4	+5.8
2	41.0	39.3	50.0	+9.0	+10.7
3	45.9	44.6	50.2	+4.3	+5.6
4	38.5	44.7	50.0	+11.5	+5.3
5	48.0	53.8	49.7	+1.7	+4.1
6	41.9	43.3	50.4	+8.5	+7.1
7	46.0	46.6	51.6	+5.6	+5.0
8	43.3	46.3	47.8	+4.5	+1.5
9	52.9	55.4	62.0	+9.1	+6.6
10	50.2	54.7	58.4	+8.2	+3.7
11	35.9	36.8	42.0	+6.1	+5.2
12	49.4	58.3	61.0	+11.6	+2.7
13	37.7	39.5	41.4	+3.7	+1.9
14	34.4	38.5	40.4	+6.0	+1.9
Averages	42.8	45.2	49.7	+6.9	+4.5

a Note that the Laplace measurements exceeded the other measurements in all cases. The conventional sinus-to-commissure technique gave the smallest diameters. The sinus-to-sinus technique gave a slightly higher measurement, as expected. The Laplace technique gave the largest measurements, as expected, exceeding the commissure-to-sinus measurement by 6.9 mm and the sinus-to-sinus measurement by 4.5 mm.

## Comment

In this study, we addressed discrepancies in the measurement of the aortic root and surgical thresholds that lead to differences in patients receiving surgery to prevent aortic dissection.

We validated the recently published, novel method of measuring the aortic root clinically, using predissection CT scans of a retrospective patient cohort. When using the Laplace diameter, rather than the commonly used sinus-to-commissure technique, more patients, 3 or 7 depending on the surgical threshold implemented (55.0 or 50.0 mm), would have qualified for surgical resection of the dilated aortic root, potentially preventing aortic dissection in their clinical course.

Although the sinus-to-sinus technique more closely approximates the Laplace measurements, for many experts, that measurement, from depth-of-sinus to depth-of-sinus, seems unnatural—not related to any “center” of the aorta. The Laplace technique establishes a center, and the Laplace radius represents the distance from that center to the largest sinus—an intuitively and mathematically sound representation of the wall tension experienced by that largest sinus.

We encourage consideration of the novel Laplace technique, which is based on precise bioengineering analysis, to enhance surgical decision-making. The ability of the novel Laplace measurement to prevent aortic dissection, shown in the present study, supports the importance and applicability of this new measurement paradigm.
